# College Students’ Knowledge, Attitudes, and Practices on Cervical Cancer Prevention in India

**DOI:** 10.7759/cureus.86429

**Published:** 2025-06-20

**Authors:** Swati Swati, Manoj Kumar, Rajan Kumar, Firdaus Bano, Bijit Biswas

**Affiliations:** 1 Obstetrics and Gynecology, All India Institute of Medical Sciences, Deoghar, IND; 2 Pediatrics, All India Institute of Medical Sciences, Patna, IND; 3 Pediatrics, All India Institute of Medical Sciences, Deoghar, IND; 4 Community and Family Medicine, All India Institute of Medical Sciences, Deoghar, IND

**Keywords:** attitudes, cervical cancer prevention, college students, human papillomavirus vaccine, india, knowledge, practices

## Abstract

Background: Cervical cancer, primarily caused by persistent infection with high-risk types of human papillomavirus, is a preventable disease. In India, it remains one of the most common cancers among women, despite significant declines in developed countries due to vaccination efforts.

Methods: This college-based cross-sectional study was conducted in Deoghar, Eastern India, among 1,156 students (1,100 from general colleges and 56 from a nursing college). A structured, self-administered questionnaire assessed socio-demographic characteristics, knowledge, attitudes, and practices regarding cervical cancer and vaccination.

Results: The median age of participants was 19 years; most were female (92.2%), Hindu (91.9%), from general academic streams (95.2%), and from rural areas (65.7%). The median knowledge score was 5 (interquartile range (IQR): 2-7). Higher scores were seen among students aged 20-21 years (p = 0.033), rural residents (p = 0.001), Muslims (p = 0.009), and nursing students (p = 0.001). Those advised by healthcare providers (p < 0.001), who believed the vaccine prevents cervical cancer (p < 0.001), and who were unconcerned about side effects (p < 0.001) also had higher knowledge. Only 2.6% were vaccinated, and they had significantly higher scores (p < 0.001). Lack of awareness (46.6%) was the most cited barrier. The internet (52.1%) and healthcare workers (22.8%) were the main information sources.

Conclusion: Although basic awareness exists, significant knowledge gaps and low vaccine uptake remain. Enhanced education, active involvement of healthcare providers, and state-led vaccination campaigns are essential to improve prevention efforts.

## Introduction

Cervical cancer is the fourth most common cancer among women worldwide. In 2022, an estimated 660,000 new cases were reported globally, with approximately 94% of the 350,000 cervical cancer-related deaths occurring in low- and middle-income countries. The highest incidence and mortality rates are observed in sub-Saharan Africa, Central America, and Southeast Asia [1]. Globally, one woman dies from cervical cancer every two minutes, with nearly 90% of these deaths occurring in resource-constrained settings [2].

In India, cervical cancer remains a major public health challenge. As of 2020, it ranked second among all cancers affecting Indian women, accounting for 18.3% of new cancer cases and 18.7% of cancer-related deaths, with a five-year prevalence rate of 18.8% [3]. Persistent infection with high-risk types of human papillomavirus (HPV)-a sexually transmitted virus-is the primary cause in nearly 99% of cervical cancer cases. The disease is largely preventable through primary prevention via vaccination and secondary prevention through screening and early treatment of precancerous lesions [4].

Recognizing its preventable nature, the World Health Organization has launched a global strategy to eliminate cervical cancer as a public health problem by 2030, emphasizing HPV vaccination, effective screening, and timely treatment [5]. Since the first HPV vaccine was introduced over a decade and a half ago, improvements in vaccine formulation, dosing schedules, and delivery strategies have enhanced accessibility [6]. Recent evidence indicates that a single-dose HPV vaccine may offer comparable protection to multi-dose regimens, providing long-term immunity and significantly reducing the burden of HPV infections and precancerous lesions [7].

Despite these advancements, uptake of the HPV vaccine remains suboptimal in many low- and middle-income countries, including India. Lack of awareness, limited access, misconceptions, and cost-related barriers contribute to low coverage. Adolescents and young adults, particularly college students, represent a crucial target group for intervention-not only due to their risk of HPV exposure but also because they are future parents and potential health advocates within their communities. Addressing their knowledge gaps and promoting positive attitudes toward vaccination and screening are essential to advance cervical cancer prevention.

This study was therefore undertaken to assess the knowledge, attitudes, and practices related to cervical cancer and its prevention among college students in Deoghar district, Jharkhand. Findings from this study are expected to inform awareness strategies and guide policy efforts to improve HPV vaccine uptake and cervical cancer control in similar settings.

## Materials and methods

Study design and setting

This college-based cross-sectional study was conducted from January to June 2023 in Deoghar district, Jharkhand, Eastern India. General colleges were purposively selected based on accessibility and institutional willingness to participate. To enable comparison between healthcare and non-healthcare students, one nursing college was also included.

Participants

The study population comprised undergraduate students enrolled in the selected institutions. All students present on the day of the survey and who provided written informed consent were eligible for inclusion. A total of 1,156 students participated in the study, including 1,100 from general academic streams and 56 from the nursing stream. Students who were absent or declined consent were excluded.

Sample size calculation

The sample size was calculated using data from a community-based study by Masood et al. [8] in Uttar Pradesh, which reported that 77.5% of participants had heard of cervical cancer. Assuming 5% absolute precision and a 95% confidence level, the minimum required sample size was calculated to be 264 using Statulator 2 (an online sample size calculator) (https://statulator.com). However, to enhance subgroup comparisons, especially between academic streams, the final sample was expanded to include 1,156 participants.

Data collection tool

A structured and pretested questionnaire, self-administered in person, was employed for data collection. The questionnaire was developed based on existing literature in consultation with experts for content and criterion validity. It captured socio-demographic information, including age, gender, religion, caste, place of residence (rural/urban), and academic stream (general or nursing). The questionnaire assessed participants’ knowledge, attitudes, and practices regarding cervical cancer and its prevention through vaccination. Additional sections explored sources of information and barriers to vaccination.

Scoring and measurement

The knowledge section comprised eight items covering awareness of cervical cancer, its viral etiology, mode of transmission, preventive measures, and vaccine efficacy. Each correct answer was scored as 1, while incorrect responses were scored as 0, yielding a total knowledge score ranging from 0 to 8. The internal consistency of the knowledge scale was high (Cronbach’s alpha = 0.856). The questionnaire also included items on attitudes (e.g., beliefs about vaccine effectiveness and safety), prior vaccination status, willingness to be vaccinated or to recommend vaccination, and reasons for non-vaccination (e.g., lack of awareness, cost, accessibility, or safety concerns).

Statistical analysis

Data were entered in Microsoft Excel (Microsoft Corp., Redmond, WA, US) and analyzed using Jamovi for Windows (Version 2.3.26) (Jamovi, Sydney, Australia). Descriptive statistics were used to summarize variables: frequencies and percentages for categorical variables and medians with interquartile ranges (IQRs) for non-normally distributed continuous variables. The cervical cancer knowledge score, found to be non-normally distributed, was compared across socio-demographic and perceptual variables using the Mann-Whitney U test (for two-group comparisons) and the Kruskal-Wallis test (for comparisons across more than two groups). Where the Kruskal-Wallis test yielded significant results, post hoc pairwise comparisons were performed using Bonferroni-adjusted Mann-Whitney U tests. A p-value of <0.05 was considered statistically significant.

## Results

Item-wise knowledge about cervical cancer and its prevention varied considerably among participants (Table 1). While nearly three-fourths were aware that cervical cancer is a leading cause of cancer-related death among Indian women, only about half knew that the vaccine has high efficacy in preventing infection. Awareness of the virus’s sexual transmission and the names of available vaccines was also limited. The median knowledge score was 5 (IQR: 2-7), reflecting moderate knowledge levels.

**Table 1:** Distribution of the study participants as per their knowledge regarding cervical cancer (n = 1,156) HPV: human papillomavirus; CI: confidence interval; K: knowledge item Data were presented as n (%) with 95% CI

Item	Variable	n (%)	95% CI
K1	Do you know cervical cancer is curable?		
	Yes	709 (61.3)	58.5-64.1
	No	447 (38.7)	35.9-41.5
K2	Do you know that cervical cancer is a leading cancer-related cause of death among Indian women?		
	Yes	809 (70.0)	67.3-72.6
	No	347 (30.0)	27.4-32.7
K3	Do you know that cervical cancer is principally caused by a virus?		
	Yes	739 (63.9)	61.1-66.6
	No	417 (36.1)	33.3-38.8
K4	Do you know that the name of the virus causing cervical cancer is HPV?		
	Yes	714 (61.8)	58.9-64.5
	No	442 (38.2)	35.4-41.1
K5	Do you have an idea that HPV spreads through sexual contact?		
	Yes	672 (58.1)	55.3-60.9
	No	484 (41.9)	39.1-44.7
K6	Do you know that this HPV infection and hence cervical cancer can be prevented by a vaccine?		
	Yes	714 (61.8)	58.9-64.5
	No	442 (38.2)	35.5-41.1
K7	Have you heard of Cervarix and Gardasil injection?		
	Yes	703 (60.8)	57.9-63.6
	No	453 (39.2)	36.4-42.0
K8	Do you know that HPV vaccine (Cervarix and Gardasil) has 95% to 100% efficacy in preventing HPV infection and hence cervical cancer?		
	Yes	566 (49.0)	46.1-51.8
	No	590 (51.0)	48.2-53.9

As shown in Table 2, knowledge scores were significantly higher among students aged 20-21 years (p = 0.033), rural residents (p = 0.001), Muslims (p = 0.009), and nursing students (p = 0.001). Better scores were also observed among those who had received a doctor’s recommendation (p < 0.001), believed in the effectiveness of the vaccine (p < 0.001), or were unconcerned about vaccine side effects (p < 0.001). Participants who had taken the vaccine, or expressed willingness to receive or recommend it, had significantly higher knowledge scores (all p < 0.001).

**Table 2:** Distribution of the study participants as per their background characteristics and knowledge regarding cervical cancer (n = 1,156) ^*^Independent sample Kruskal-Wallis test ^#^Mann-Whitney U test OBC: other backward caste; SC: scheduled caste; ST: scheduled tribe; HPV: human papillomavirus; IQR: interquartile range Data were represented as n (%) and median (IQR)

Variable	n (%)	Knowledge score (median, IQR)	p-value
Age in completed years			
<18	114 (9.9)	5, 2-7	0.033^*^
18-19	491 (42.5)	5, 2-7	
20-21	346 (29.9)	6, 3-7	
>21	205 (17.7)	5, 2-7	
Gender			
Female	1,066 (92.2)	5, 3-7	0.058^#^
Male	90 (7.8)	4, 2-6	
Residence			
Rural	760 (65.7)	5, 3-7	0.001^#^
Urban	396 (34.3)	5, 2-7	
Religion			
Muslim	94 (8.1)	6, 4-7	0.009^#^
Hindu	1,062 (91.9)	5, 2-7	
Caste			
General	444 (38.4)	5, 2-7	0.576^*^
OBC	576 (49.8)	5, 3-7	
SC	80 (6.9)	6, 2-7	
ST	56 (4.8)	5, 2-7	
Stream			
Nursing	56 (4.8)	6, 5-7	0.001^#^
General	1,100 (95.2)	5, 2-7	
Advised by doctor or health worker for HPV vaccine (n = 1,066)			
Yes	396 (37.1)	6, 5-8	<0.001^#^
No	670 (62.9)	4, 2-6	
Thought that HPV vaccine can effectively prevent cervical cancer			
Yes	823 (71.2)	6, 4-8	<0.001^#^
No	333 (28.8)	2, 0-5	
Thought that one should not get concerned about side effects of HPV vaccine			
Yes	700 (60.6)	6, 4-7	<0.001^#^
No	456 (39.4)	4, 0-6	
Have taken HPV vaccine (n = 1,066)			
Yes	28 (2.6)	7, 6-8	<0.001^#^
No	1,038 (97.4)	5, 2-7	
Willingness to get HPV vaccination after the study (n = 1,038)			
Yes	559 (53.9)	6, 4-7	<0.001^#^
No	479 (46.1)	4, 1-6	
Willingness to recommend HPV vaccination to others			
Yes	928 (80.3)	6, 3-7	<0.001^#^
No	228 (19.7)	5, 2-7	

Despite this (n = 1,066), vaccine uptake was extremely low, with only 28 (2.6%) participants reporting having received it. Vaccination rates did not differ significantly between nursing (3.6%) and general (2.6%) students (p = 0.650). Of those, most had completed the full dose regimen. Among unvaccinated individuals, willingness to receive the vaccine in the future was reported by over half.

**Figure 1:**
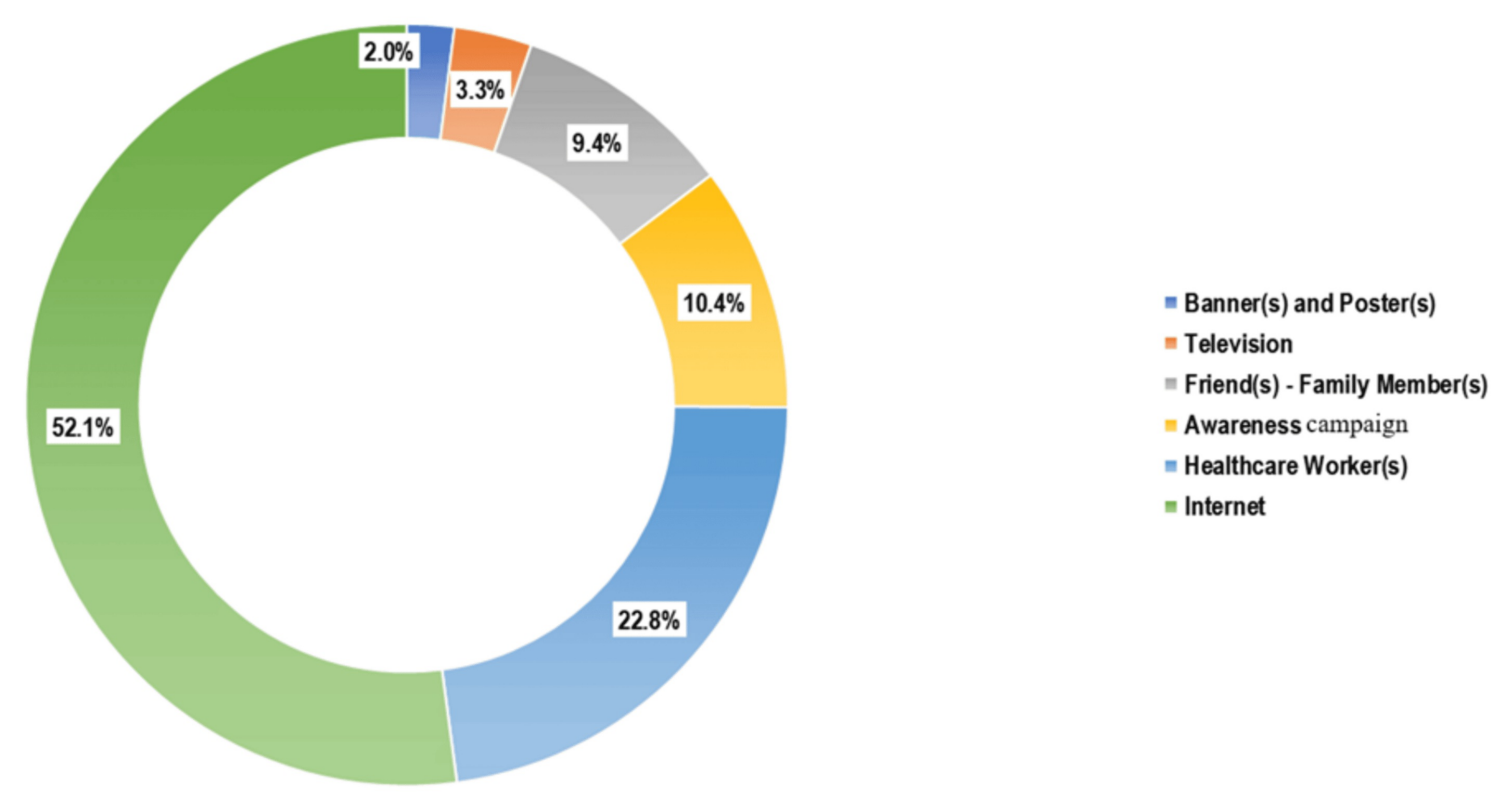
Distribution of study participants by the predominant source of knowledge regarding HPV vaccine (n = 714) HPV: human papillomavirus

**Figure 2:**
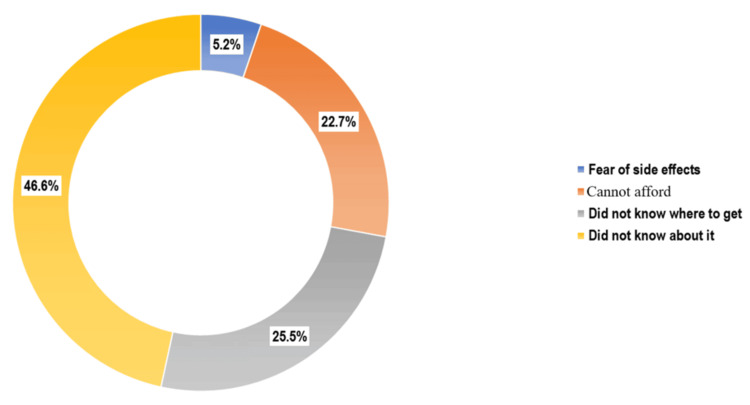
Distribution of study participants by predominant reasons for not receiving the HPV vaccine (n = 1,038) HPV: human papillomavirus

Figure 1 illustrates (n = 714) that the internet was the most common source of information (372 (52.1%)), followed by healthcare workers (163 (22.8%)) and awareness campaigns (74 (10.4%)). As shown in Figure 2 (n = 1,038), the predominant reason for non-vaccination was the lack of awareness (484 (46.6%)), followed by uncertainty about where to obtain the vaccine (265 (25.5%)), cost-related concerns (236 (22.7%)), and fear of side effects (54 (5.2%)).

## Discussion

This study evaluated the knowledge, attitudes, and practices regarding cervical cancer prevention among college students in Deoghar district, Jharkhand, using a cross-sectional design. The median knowledge score was five out of eight, reflecting moderate awareness with variation across specific items. Knowledge levels were significantly higher among older students, rural residents, Muslims, and nursing students. Despite this, vaccine uptake was extremely low, with only about one in 40 participants reporting receipt of the HPV vaccine. Lack of awareness was the predominant barrier, and the internet emerged as the main source of information, followed by healthcare providers.

India is home to the second-largest tribal population globally, following Africa, with scheduled tribes (ST) constituting 8.6% of the national population [9]. In Jharkhand, this proportion is significantly higher-26.2%-placing it among the top states in terms of tribal concentration [10]. In Deoghar district specifically, tribal communities represent about 12.1% of the population [11]. In our study, 4.8% of participants identified as ST, which is lower than the district average, possibly due to lower enrollment of tribal youth in higher education institutions. Given the limited data on awareness of cervical cancer and vaccination in Jharkhand, particularly among young adults, this study was conducted to address that gap by assessing college students in Deoghar.

The median age of participants in our study was 19 years, comparable to university-based studies in Indonesia (23 years) [12] and Hong Kong (19 years) [13]. The female predominance (over 90%) in our sample aligns with the all-female sample in the Hong Kong study [13]. About two-thirds of the participants in our study resided in rural areas, closely matching the urban-rural distribution reported in the 2011 census for Deoghar [11]. Religious distribution in our sample was also largely representative, although the proportion of Muslim students (8.1%) was slightly higher than the district's average (4.96%). Most participants (around 95%) belonged to general academic streams, with a smaller proportion from nursing, which was intended to enable stream-based comparisons.

Participants showed moderate knowledge of cervical cancer and HPV-related items, with correct responses ranging from 49.0% to 70.0%, and a median score of 5 out of 8. This level of awareness is similar to that reported in Indonesian college populations [12], and item-wise scores were also comparable to those in Hong Kong, where knowledge levels varied between 22% and 76.9% [13]. Awareness that cervical cancer is vaccine-preventable was observed in 61% of participants in our study, higher than the 42% reported in an earlier Italian study [14], but still lower than ideal. Notably, only 58% of our participants were aware that HPV is transmitted through sexual contact, in contrast to 75%-90% reported in past Italian studies [14,15].

The internet was the predominant source of information for over half of the participants (Figure 1), a pattern also observed in Hong Kong [13]. In contrast, television played a limited role in our setting (3.3%), unlike in the Hong Kong study, where it was a leading source [13]. One likely reason is that Indian college students may have greater access to internet-enabled smartphones than to scheduled television viewing. Healthcare providers were cited as a source by about one-fourth of our participants, which is lower than the 60% reported in an earlier Italian study [15]. Still, over one-third had been advised to take the vaccine by a doctor or health worker, compared to only 8.2% in the Hong Kong sample [13]. These findings underscore the untapped potential of healthcare providers in vaccine advocacy across Indian campuses.

Only 2.6% of participants in our study reported having received the HPV vaccine. This figure is far below that of Sikkim, a northeastern Indian state that achieved over 95% coverage through government-led campaigns [16], and also lower than college-based studies in Odisha, where partial uptake exceeded 50% despite high willingness [17]. Limited coverage is consistent with broader regional data; across South Asian countries, pooled HPV vaccine uptake remains low at approximately 8% [18]. Factors such as lack of national immunization programs, cost barriers, and limited awareness are likely contributors.

Among unvaccinated students in our study, the most commonly cited barrier was lack of awareness (46.6%), followed by uncertainty about where to obtain the vaccine (25.5%), affordability (22.7%), and concerns about side effects (5.2%) (Figure 2). Similar concerns have been documented elsewhere-70% of Hong Kong students found the vaccine too expensive, and 11% were unsure where to get it [13]. Fear of side effects was noted by 39% in our sample, closely mirroring the 45% reported in Hong Kong [13], which is consistent with international literature where perceived vaccine safety remains a deterrent [14,15]. In our study, 28% of students expressed doubts about vaccine efficacy, similar to the skepticism seen among Italian parents who questioned its role in preventing HPV infection [15].

This study has certain limitations that must be acknowledged. Firstly, the use of purposive sampling and self-reported data may introduce selection bias and social desirability bias, potentially affecting the accuracy of responses related to sensitive topics such as vaccine uptake and sexual transmission. Secondly, the study was limited to undergraduate students from selected colleges in a single district of Jharkhand, which may restrict the generalizability of the findings to other populations, particularly non-college-going youth or those from different geographic or socio-cultural contexts. Thirdly, the cross-sectional design captures knowledge and practices at a single time point and does not allow for causal inference or assessment of changes over time. Lastly, as most participants were from general academic streams with only a small proportion from nursing backgrounds, comparisons between healthcare and non-healthcare students should be interpreted with caution.

## Conclusions

This study found that while general awareness of cervical cancer was present among college students in Deoghar, knowledge about its viral cause, sexual transmission, and prevention through vaccination was limited. Vaccine uptake was very low, with the most commonly cited barriers being lack of awareness, not knowing where to get vaccinated, cost concerns, and fear of side effects. Higher knowledge scores were observed among older students, rural residents, Muslims, nursing students, and those who had been advised by healthcare providers. These findings suggest the need to strengthen cervical cancer and vaccination-related awareness within college settings, especially in general academic streams. Engagement of healthcare providers in delivering vaccine-related information and addressing misconceptions may help improve awareness and acceptance.

## Appendices

**Table 3:** Cervical cancer prevention knowledge assessment questionnaire HPV: human papillomavirus; K: knowledge item

Item	Questions	Responses	Score
K1	Do you know cervical cancer is curable?	Yes	1
		No	0
K2	Do you know that cervical cancer is a leading cancer-related cause of death among Indian women?	Yes	1
		No	0
K3	Do you know that cervical cancer is principally caused by a virus?	Yes	1
		No	0
K4	Do you know that the name of the virus causing cervical cancer is HPV?	Yes	1
		No	0
K5	Do you have an idea that HPV spreads through sexual contact?	Yes	1
		No	0
K6	Do you know that this HPV infection and hence cervical cancer can be prevented by a vaccine?	Yes	1
		No	0
K7	Have you heard of Cervarix and Gardasil injection?	Yes	1
		No	0
K8	Do you know that HPV vaccine (Cervarix and Gardasil) has 95% to 100% efficacy in preventing HPV infection and hence cervical cancer?	Yes	1
		No	0
